# Effects of the herbicide 2,4 D active ingredient and commercial formulations on the early life stages of the amphibian *Physalaemus gracilis*

**DOI:** 10.1007/s10646-026-03094-9

**Published:** 2026-06-17

**Authors:** Cristina Bridi, Guilherme Felicioni, Jenifer Eduarda Luterek, Inete Cleide Baú, Flavia Bernardo Chagas, Aline Pompermaier, Carla Alves, Paulo Afonso Hartmann, Marilia Hartmann

**Affiliations:** 1 Laboratory of Ecology and Conservation, Federal University of Fronteira Sul, Erechim, RS Brazil; 2 Fisheries Institute, São Paulo, SP Brazil; 3 Federal Institute of Education, Science and Technology of Rio Grande do Sul, Sertão, RS Brazil

**Keywords:** 2,4-dichlorophenoxyacetic acid, Anura, ecotoxicology, hatchling, pesticide

## Abstract

The herbicide 2,4-D is extensively employed and detected in surface waters, representing a risk for amphibians. Species of the genus *Physalaemus*, such as *P. gracilis*, show sensitivity to the compound even at low concentrations. This study compared the toxicity of the active ingredient (D_AI_) and two of its commercial formulations of 2,4-D (DBH_1_ and DBH_2_) on survival, heart rate, swimming activity, and biochemical parameters (TBARS levels and CAT activity) of *P. gracilis* during its initial development stages. The tested concentrations (5, 10, 15, 20, and 25 µg/L) were selected based on the limits established for 2,4-D in Brazilian freshwater regulations. D_AI_ decreased survival and altered swimming activity at all tested concentrations, increased TBARS levels at 5 and 10 µg/L, and reduced CAT activity at 20 and 25 µg/L. Both commercial formulations also affected survival and swimming activity at all concentrations; DBH_1_ reduced heart rate at 15 and 25 µg/L, while DBH_2_ increased it at all tested concentrations. Additionally, DBH_1_ increased TBARS levels at 15 µg/L in exposed embryos and tadpoles, whereas DBH_2_ decreased TBARS levels at 5 µg/L and reduced CAT activity at all tested concentrations. Overall, exposure to 2,4-D and its commercial formulations induced physiological and behavioral alterations in *Physalaemus gracilis* tadpoles, although the magnitude and direction of the effects varied among compounds.

**Supplementary Information:**

The online version contains supplementary material available at 10.1007/s10646-026-03094-9.

## Introduction

The expansion of large-scale agro-industrial production drives the continuous use of pesticides (Gunderson et al. 2025), with herbicides being the most commonly applied. This widespread use leads to environmental accumulation, especially in aquatic systems near agricultural areas (Parven et al. 2025). Among them, 2,4-D (2,4-Dichlorophenoxyacetic acid), first commercialized in the 1940s, is found in over 1,500 commercial products (Sun et al. 2020) and has been the most widely used herbicide worldwide over the past decade (Mathur 2025), ranking second in sales among pesticides in Brazil (Souza et al. 2023; Ibama 2024). While primarily applied to wheat and soybean, 2,4-D is also be used in rice, sugarcane, corn, coffee, sorghum, and pastures (Barcellos 2025). Its intense use is justified by its low cost, effectiveness, and selectivity in controlling weeds (Dehnert et al. 2018). However, it also presents environmental risks due to its capacity to infiltrate the soil and reach surface waters and groundwater through runoff (Ighalo et al. 2023).

The World Health Organization classified 2,4-D as moderately hazardous, whereas the United States Environmental Protection Agency (USEPA 2026) considered the product slightly to moderately toxic, and the Brazilian National Health Surveillance Agency (ANVISA, 2023) classified it as extremely toxic. For human health, occupational exposure to 2,4-D can cause skin and eye irritation, coughing, and respiratory difficulties (Demissie et al. 2022). Acute ingestion is associated with gastrointestinal symptoms, but cardiovascular and even neurological effects may also occur (Bradberry et al. 2000). The herbicide can also persist in the soil and negatively affect non-target plants by interfering with their growth and physiological processes, potentially leading to a substantial reduction in plant diversity (Islam et al. 2018). Furthermore, 2,4-D not only reaches surface and groundwater but also exhibits moderate persistence in aquatic environments, with a half-life in water ranging from 15 to 90 days (Girardi et al. 2013), which may affect the aquatic organisms inhabiting these environments.

Although 2,4-D has been banned in ten countries (PAN 2024), its use remains permitted in Brazil. In 2019, it was temporarily prohibited in Rio Grande do Sul, southern Brazil, due to complaints of product misapplication and a lack of inspectors as a consequence of a strike (SEAPDR 2019). In September 2025, the Court of the State of Rio Grande do Sul prohibited the use of 2,4-D for 120 days due to damages caused by pesticide drift on broadleaf crops, such as vineyards and apple orchards (TJRS 2025). However, this restriction was later suspended within the same month due to potential economic and administrative impacts (Meirelles 2025). Despite these measures, 2,4-D continues to be widely used in the region and exhibits high volatility, resulting in drift that can extend 200 to 500 m from the application site (Costa et al. 2012; Gregorio et al. 2015), highlighting its potential to reach nearby aquatic environments.

The presence of 2,4-D in surface waters has been reported in several countries, including Greece (0.752 µg/L; Papadakis et al. 2015), Hungary (1003 ng/L; Székács et al. 2015), and Portugal (967.47 ng/L, Palma et al. 2014) in Europe; China (66.2 ng/L, Sun et al. 2020) and Malaysia (329.42 µg/L, Ismail et al. 2015) in Asia; and Argentina (0.253 µg/L, Pérez et al. 2021), the United States (11.5 µg/L, Ensminger et al. 2012) and Brazil in the Americas. In Brazil, 2,4-D was detected in surface waters in most States, mainly in highly agricultural regions (Brovini et al. 2021). The highest concentration detected in Brazilian surface waters was in Rio Grande do Sul, with 90 µg/L (Lima et al. 2020).

Aquatic environments are essential for the reproduction and development of most anuran species, and the presence of pesticides in surface waters is the main route of exposure to toxic products for amphibian species. Amphibians have been facing a great decline since the last century (Toledo et al. 2023), with more than 40% of the species threatened with extinction (IUCN 2025). The main causes of the decline are associated with anthropogenic impacts on nature, including modern agriculture (Renoirt et al. 2024), and the use of pesticides (Agostini et al. 2020; Wanger et al. 2023). In the vast majority of anuran species, the embryonic and larval stages occur in freshwater habitats, during which development is particularly vulnerable to disturbances from contaminants present in the water (Orton and Tyler 2015).

Anurans serve as model organisms to investigate the effects of contaminants in their different development stages (Ferreira et al. 2025). The group is considered an environmental indicator because its members can absorb contaminants through their skin and gills (Amaral et al. 2019). In this context, native species are particularly relevant because they are closely integrated with their local environment (Wingen et al. 2023). The genus *Physalaemus*, which belongs to the family Leptodactylidae, is a large genus of small frogs comprising approximately 50 described species. Commonly known as dwarf frogs or foam frogs, this genus is distributed throughout Latin America (Heyer and Crombie 2005; Frost 2024). Studies investigating the toxicity of 2,4-D in *Physalaemus cuvieri* have shown that even at low concentrations this compound can cause mortality (53.5, 75 and 100 µg/L), morphological anomalies (100 µg/L), and nuclear abnormalities (30–100 µg/L) in tadpoles (Santos et al. 2024), as well as weight loss at concentrations ranging from 1 to 50 µg/L (Folador et al. 2025). Other species of *Physalaemus* have also shown susceptibility to 2,4-D exposure. In tadpoles of *P. albonotatus*, concentrations of 0.75, 1.5, and 3.0 mg/L induced several histological alterations in the liver tissue, including hepatocyte vacuolization, sinusoidal enlargement, dilation of blood vessels, and an increase in melanin-containing macrophages (Curi et al. 2019). Exposure to the herbicide accelerated the metamorphosis of *Physalaemus centralis*, particularly at a concentration of 257.88 mg/L, a response possibly triggered by physiological stress (Figueiredo and Rodrigues 2014).

*Physalaemus gracilis*, popularly known as the ‘Graceful Dwarf Frog’ is a native species found in southern Brazil, Uruguay, and northeastern Argentina, and possibly in southeastern Paraguay (Frost 2024). Classified as ‘Least Concern’ by the International Union for Conservation of Nature (IUCN 2025), its serves as an environmental bioindicator for contaminants like pesticides (Hartmann et al. 2023). The species is particularly useful because its foam nests are easily identifiable and collectible, being deposited at the edge of streams and various water bodies, including permanent and temporary ponds (Duarte et al. 2022).

Ponds are important zones for pesticide dissipation and can intercept runoff from agricultural areas (Imfeld et al. 2021), potentially exposing amphibians that reproduce in these environments, such as *Physalaemus gracilis*. Despite the widespread use of 2,4-D, information on the effects of its commercial formulations compared with the active ingredient on native amphibian species remains limited. Therefore, this study aimed to evaluate and compare the toxicity of the active ingredient and two commercial formulations of 2,4-D by assessing survival, heart rate, swimming activity, and biochemical parameters during the early developmental stages of *Physalaemus gracilis*.

## Materials and methods

### Chemical substances

For the exposure to the active ingredient (D_AI_), we used 2,4-D Pestanal^®^ with 98% purity, dissolved in 0.2% of Dimethyl sulfoxide (DMSO). Two commercial formulations of 2,4-D-based herbicide (DBHs) were compared: (1) Commercial formulation 1: DBH_1_ – composed of 806 g/L of 2,4-dichlorophenoxyacetic acid, 670 g/L acid equivalent and 380.9 g/L of other unspecified ingredients, registered at the Ministry of Agriculture and Livestock of the Brazil (MAPA) under n° 01803; (2) Commercial formulation 2: DBH_2_ - composed of the same quantity of 2,4-D as DBH_1_, that is, of 806.0 g/L of dimethylamine salt of (2,4-dichlorophenoxy), 670.0 g/L acid equivalent, but with 526.6 g/L of other ingredients, registered at MAPA under n° 30009. Both commercial formulations are classified as Environmental Hazard Class III, indicating products that are toxic to the environment.

The concentrations used in this experiment were based on Brazilian legislation for surface waters, specifically Resolution No. 357 of the National Council for the Environment (Brazil 2005). Five concentrations were chosen (5, 10, 15, 20, and 25 µg/L), representing intermediate values between the maximum permitted limit for Class I and II freshwater (4 µg/L) and the established for Class III freshwater (30 µg/L).

### Test organism

A single clutch of eggs of *P. gracilis* were collected with less than 24 hours post-oviposition from a non-agricultural pond (Latitude: -27º43’ 46.11” S; Longitude: -52º16’ 54.40” W) and taken to the Ecology and Conservation Laboratory, where they were placed in aquaria with reconstituted water (FETAX solution, composed of mineral salts such as NaCl, NaHCO₃, KCl, CaCl₂, MgSO₄, and CaSO₄, standardized according to ASTM 1439-12).

### Exposure

Shortly after collection, the eggs were separated, and fecundity was determined under a stereomicroscope. Viable eggs were transferred individually to 24-well culture plates, with each well containing 3 mL of solution. The experimental design allocated four wells per plate as controls (FETAX solution only) and 20 wells for exposure to FETAX medium containing the target 2,4-D concentration. All tested concentrations and substances were assessed in triplicate, in addition to three plates containing only FETAX as a control. To evaluate potential toxicity, an additional control group was included containing 0.2% DMSO, corresponding to the same concentration used in the experimental solution of the active ingredient (D_AI_). This solvent control was employed to evaluate potential effects of the carrier used to dissolve D_AI_. The DMSO concentration applied in this study corresponds to approximately 6–9% of the reported 96-h LC₅₀ values for amphibian embryos (2.19–3.19%; Coelho et al. 2026).

The assay was conducted under static conditions and lasted seven days. A seven-day static exposure was adopted based on the half-life of 2,4-D in water (7–10 days), as well as evidence indicating that measured concentrations remain close to nominal levels over several days in experimental systems (Mesak et al. 2018; Martínez-Ruiz and Martínez-Jerónimo, 2018; Velásquez et al. 2025; White et al. 2022). In addition, water renewal was avoided to minimize handling stress to the tadpoles, which are particularly sensitive during early developmental stages. At the beginning of the experiment, the embryos were between developmental stages (S) 12 and 18, and by the final day they had reached the beginning of S25, considered the hatchling stage according to Gosner (1960). In this study, the term “embryos” was used to refer to the organisms at the start of the assay, whereas “tadpoles” was used for the subsequent stages, corresponding to S25 at the end of the experiment.

### Survival

The embryos and tadpoles were observed individually every day under a stereomicroscope to check the survival, and dead individuals were removed from the wells.

### Heart rate

The heart rate of the tadpoles was measured on the seventh day of the experiment using 20 individuals from each concentration. They were placed individually in a ventral position under the stereomicroscope and monitored for 60 s (Pompermaier et al. 2024).

### Swimming activity

On the last day of the assay (seventh day), swimming activity was visually observed. Following a stimulus with a glass rod, all surviving tadpoles were classified as: (1) normal, swimming freely throughout the well; (2) lethargic, swimming less frequently; (3) spasmodic, exhibiting spasms in response to a stimulus; and (4) hyperactive, showing increased swimming activity in response to the applied stimulus (Rutkoski et al. 2020). Because the number of survivors varied across concentrations, the data were transformed into proportions relative to the number of live individuals in each treatment for statistical analyses. This allowed for the standardization of response variables across concentrations.

### Biochemical parameters

For the biochemical analyses, 20 tadpoles were selected per group, with three replicates per treatment. The number of animals included in the biochemical analyses was defined based on survivors at the end of the assay, thereby ensuring uniformity in sample size among groups. This sample size is consistent with that reported by Pompermaier et al. (2024) and Folador et al. (2026). Tissues samples were prepared as a single homogenate, in which whole tadpoles were pooled and homogenized on ice with Tris-HCl buffer (50 Mm, pH 7.4) for one minute using a Potter homogenizer. The homogenate was then centrifuged at 7000 × g for 10 min at 4 °C. The resulting supernatant was collected for analysis, and the pellet was discarded. All procedures were carried out on ice.

The analysis process for Catalase (CAT) enzyme activity followed the methodologies of Johansson and Borg (1988) and Góth (1991). The samples were evaluated using a HIDEX microplate reader (Hidex Chameleon, Berlin, Germany). CAT activity was expressed in CAT units per mg of protein^− 1^ and measured at 240 nm.

To quantify malondialdehyde (MDA) levels by thiobarbituric acid reactive substances (TBARS), the methods described by Moore and Roberts (1998) were followed. For the reaction, 80 µL of the sample were combined with 160 µL of trichloroacetic acid (TCA 10%). Then, the samples were centrifuged at 13,000 × g at 4 °C for 10 min. After this process, 100 µL of the supernatant were removed and mixed with 1400 µL of thiobarbituric acid (TBA 0.67%). The mixtures were then heated to 95 °C for 30 min. Absorbance was determined at 532 nm, and the final MDA levels were obtained from a standard curve (0–3 mM), expressed as nmol of MDA per mg of protein.

### Data analyses

For survival analysis, the Kaplan-Meier method was used. Data were analyzed for normality using the Kolmogorov-Smirnov test and for homogeneity using Bartlett’s test. Parametric data were analyzed using One-Way ANOVA followed by Tukey’s post-hoc test, and non-parametric data were analyzed using the Kruskal-Wallis test followed by Dunn’s post-hoc test. Data significance was considered when *p* < 0.05. All analyses were performed using GraphPad Prism software.

## Results

### Survival

**Fig. 1:**
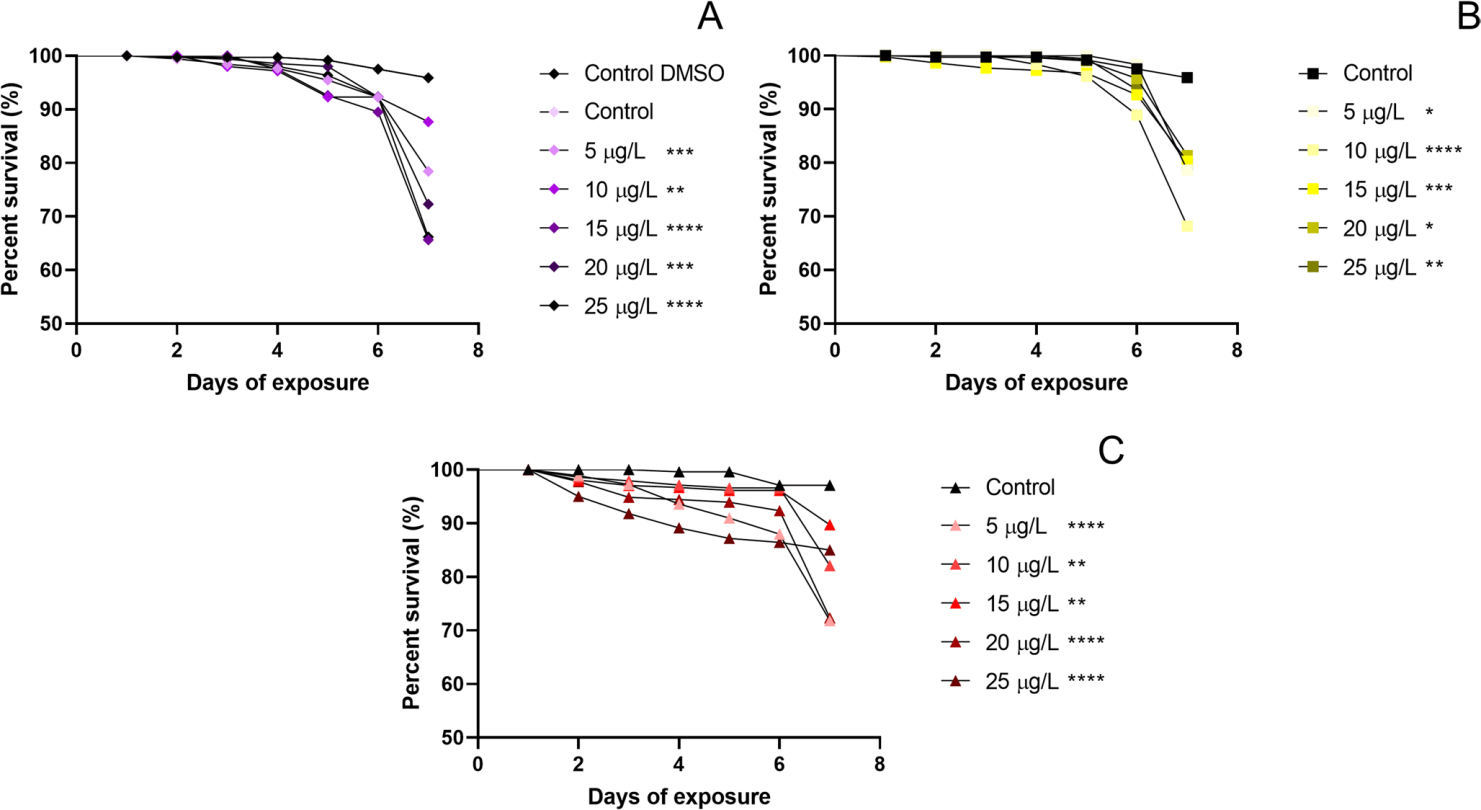
Survival of *Physalaemus gracilis* embryos and tadpoles exposed to two commercial formulations and the active ingredient of the herbicide 2,4-D for seven days. Data represent the percentage of surviving individuals over time. Survival distributions were compared using the log-rank (Mantel-Cox) test following Kaplan-Meier estimation. (A) Active ingredient (D_AI_); (B) Commercial formulation 1 (DBH_1_); (C) Commercial formulation 2 (DBH_2_). Asterisks indicate statistical differences from the control (*p < 0.05; **p < 0.01; ***p < 0.001; ****p < 0.0001)

The D_AI_, DBH_1_, and DBH_2_ of 2,4-D affected the survival of *P. gracilis* at all concentrations tested (D_AI_: χ² = 24.89; *p* = 0.0001; Fig. 1A; DBH_1_: χ² = 24.19; *p* = 0.0002; Fig. 1B; DBH_2_: χ² = 43.61; *p* < 0.0001; Fig. 1C). No significant differences were observed between the DMSO control and the FETAX-only control (χ² = 0.001; *p* > 0.9997; Fig. 1A).

### Heart rate

**Fig. 2:**
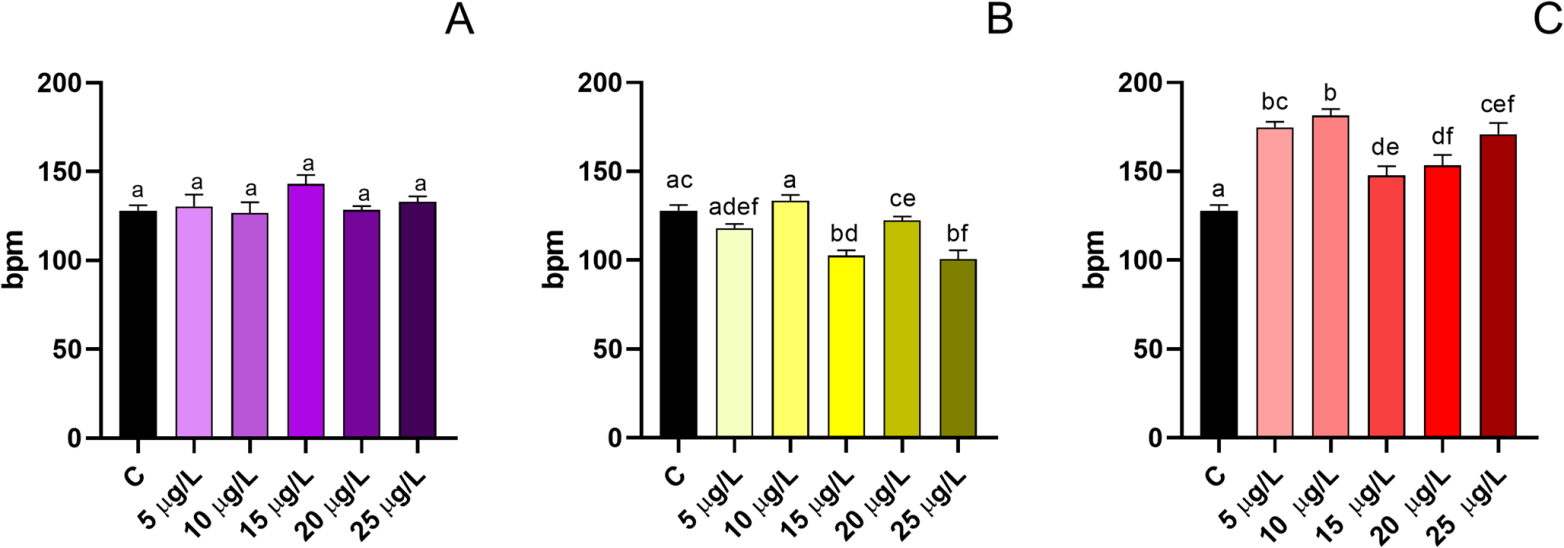
Heart rate (beats per minute, bpm) in *Physalaemus gracilis* tadpoles exposed to the active ingredient and two commercial formulations of 2,4-D after seven days of exposure. (A) Active ingredient (D_AI_); (B) Commercial formulation 1 (DBH_1_); (C) Commercial formulation 2 (DBH_2_). Data are expressed as mean ± SEM. Different letters refer to significant differences by the Dunn test (*p* < 0.05)

Exposure to D_AI_ did not affect the heart rate of tadpoles (H_(6,160)_ = 6.881; *p* = 0.2297; Fig. 2A), whereas both commercial formulations (DBHs) had significant effects. The DBH_1_ decreased heart rate at concentrations of 15 and 25 µg/L (H_(6,159)_ = 46.85; *p* < 0.0001; Fig. 2B), while DBH_2_ increased heart rate at all tested concentrations (F_(6,155)_ = 26.80; *p* < 0.0001; Fig. 2C).

### Swimming activity

**Fig. 3:**
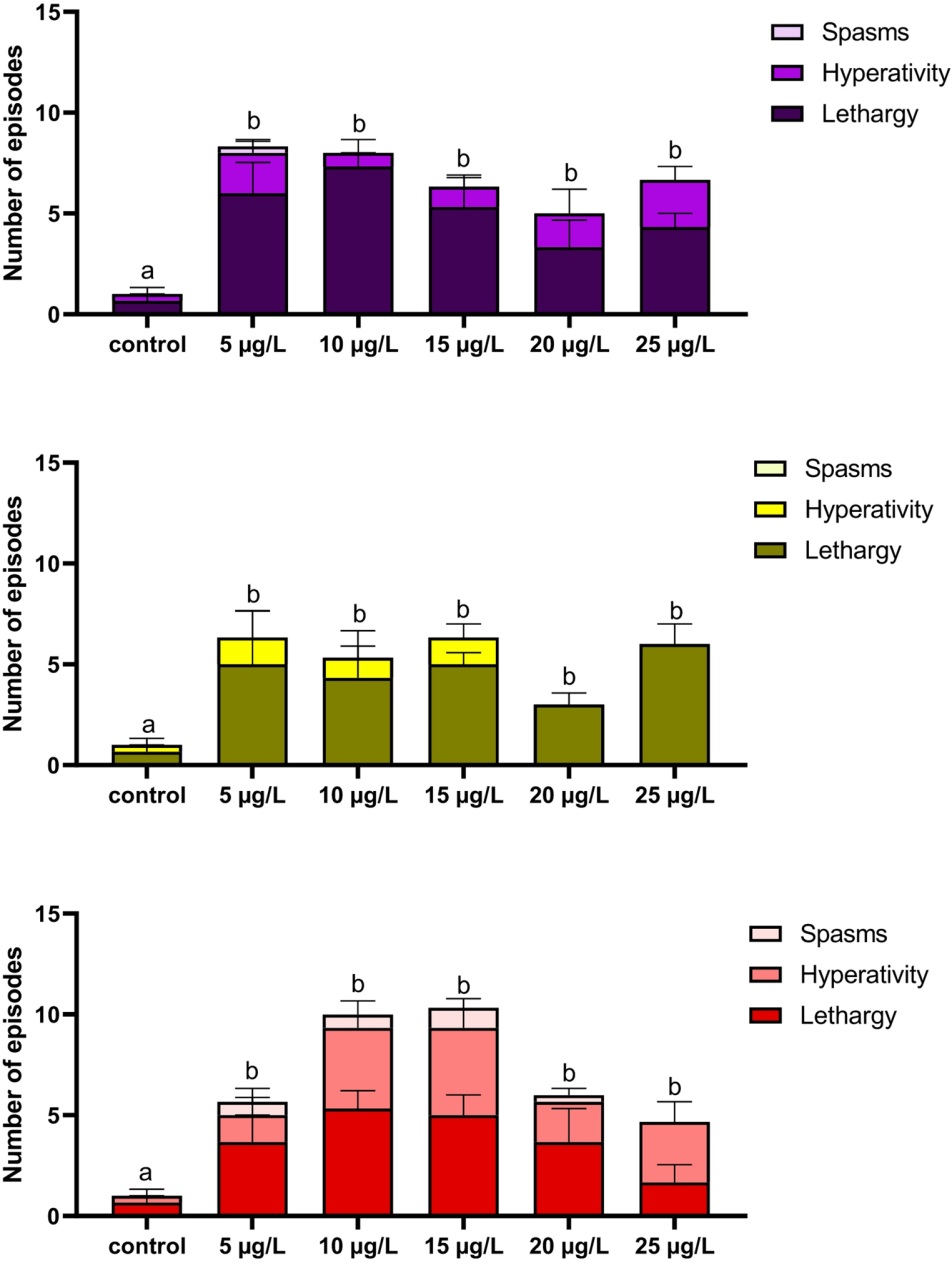
Number of *Physalaemus gracilis* tadpoles exhibiting irregular swimming activity after seven days of exposure to the active ingredient and two commercial formulations of 2,4-D. (A) Active ingredient (D_AI_); (B) Commercial formulation 1 (DBH_1_); (C) Commercial formulation 2 (DBH_2_). Data are expressed as mean ± SEM. Different letters refer to significant differences by the Dunn test (*p* < 0.05)

Both the active ingredient (D_AI_) and the commercial formulations (DBHs) of 2,4-D affected the swimming activity of tadpoles, resulting in increased irregular swimming (D_AI_: H_(6,246)_ = 34.77; *p* < 0.0001; Fig. 3A; DBH_1_: H_(6,179)_ = 43.18; *p* < 0.0001; Fig. 3B; DBH_2_: H_(6,205)_ = 63.07; *p* < 0.0001; Fig. 3C). The most common alteration across all concentrations and formulations was lethargy, followed by hyperactivity and spasms.

### Biochemical parameters

**Fig. 4:**
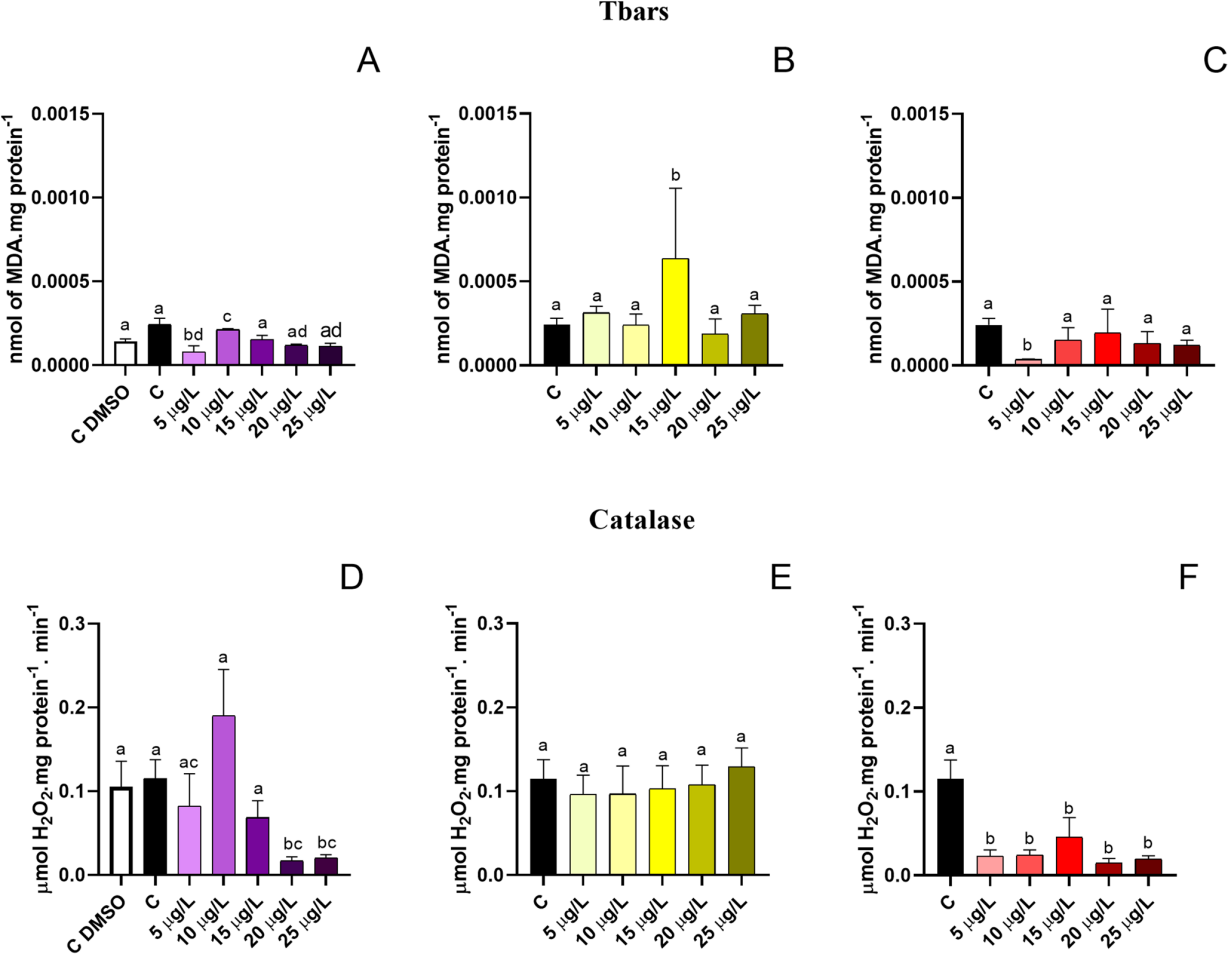
Quantification of TBARS (A, B, C) and catalase (CAT) activity (D, E, F) in *Physalaemus gracilis* tadpoles exposed for seven days to different concentrations of 2,4-D. The graphs represent: (A, D) active ingredient (D_AI_), (B, E) commercial formulation 1 (DBH_1_); and (C, F) commercial formulation 2 (DBH_2_). Data are expressed as mean ± SD. Different letters refer to significant differences by the Dunn or Tukey test (*p* < 0.05)

TBARS levels were altered by exposure to D_AI_, with a decrease at 5 µg/L and an increase at 10 µg/L (F_(5,30)_ = 12.02; *p* < 0.0001; Fig. 4A). For DBH_1_, exposure increased levels at the concentration of 15 µg/L (F_(5,30)_ = 3.13; *p* = 0.021), and for DBH_2_ it decreased at 5 µg/L (F_(5,19)_ = 3.473; *p* = 0.0213). CAT enzyme activity was reduced following exposure to D_AI_ at concentrations of 20 and 25 µg/L (H_(6,49)_ = 18.10; *p* = 0.0028; Fig. 4D) and was decreased by DBH_2_ at all concentrations (H_(6,35)_ = 14.41; *p* = 0.0132; Fig. 4F). DBH_1_ did not affect CAT activity (F_(6,43)_ = 0.2512; *p* = 0.9366; Fig. 4E).

In summary, one or more concentrations of D_AI_ decreased survival, altered swimming activity and TBARS levels, and reduced CAT activity. Similarly, DBHs also affected survival and swimming activity; however, DBH_1_ reduced heart rate and increased TBARS in exposed embryos and tadpole, whereas DBH_2_ increased heart rate and decreased both TBARS levels and CAT activity. This summary of results is represented in Table 1.

**Table 1:** Summary of effects after 7 days of exposure to the active ingredient (D_AI_) and commercial formulations (DBHs) of 2,4-D in *Physalaemus gracilis*: (↓) decreased, (↑) increased, (-) no effect

2,4-D	Group (µg/L)	Survival	Heart rate	Irregular swimming	TBARS	CAT
D_AI_	5	↓	-	↑	↓	**-**
	10	↓	-	↑	↑	**-**
	15	↓	-	↑	-	**-**
	20	↓	-	↑	-	↓
	25	↓	-	↑	-	↓
DBH_1_	5	↓	-	↑	-	**-**
	10	↓	-	↑	-	**-**
	15	↓	↓	↑	↑	**-**
	20	↓	-	↑	-	**-**
	25	↓	↓	↑	-	**-**
DBH_2_	5	↓	↑	↑	↓	↓
	10	↓	↑	↑	-	↓
	15	↓	↑	↑	-	↓
	20	↓	↑	↑	-	↓
	25	↓	↑	↑	-	↓

## Discussion

This study provides evidence that both the active ingredient (D_AI_) and the commercial formulations (DBH_1_ and DBH_2_) of 2,4-D impair the early life stages of *P. gracilis*. Effects were detected at concentrations compliant with Brazilian legislation (Brazil 2005) and even below the maximum permissible levels in other countries where the species is found, such as Argentina (100 µg/L; Argentina 2023) and Uruguay (30 µg/L for drinking water, UNIT 2010).

Regarding survival, studies with other species of *Physalaemus* reported effects of 2,4-D at concentrations above 52.5 µg/L for *P. cuvieri* (Santos et al. 2024), 257.77 mg/L (25,7770 µg/L), for *P. centralis* (Figueiredo and Rodrigues 2014) and 43.7 mg/L (43,700 µg/L), for *P. albonotatus* (Curi et al. 2019). In other amphibian species, survival was not as severely affected, even at higher concentrations of 2,4-D. For example, in tadpoles of *Xenopus laevis* (up to 95,500 µg/L; Coady et al. 2013), *Rhinella marina* (up to 70,710 µg/L; Figueiredo and Rodrigues 2014), *Lithobates catesbeianus* (up to 350,000 µg/L; Viriato et al. 2021), *Leptodactylus fuscus* (up to 448.74 µg/L; Freitas et al. 2022), *Scinax squalirostris* (up to 30 µg/L; Wingen et al. 2023), *Boana plantanera* and *Engystomops pustulosus* (up to 211,070 µg/L and 167,520 µg/L; Velásquez and Bautista 2025 . Most of these species were exposed to different commercial formulations of 2,4-D; for example, *P. cuvieri* was exposed to DBH_2_ (Santos et al. 2024), whereas Folador et al. (2025, 2026 ) used DBH_1_. Neither formulation caused changes in survival. All of these studies were conducted with fully developed tadpoles (stage S25); therefore, it remains unclear whether the observed differences in sensitivity are due to species-specific traits or to life stage. Overall, in terms of survival, *P. gracilis* appears to be the most sensitive species identified to date to 2,4-D exposure.

The increase in heart rate is considered an energy-reallocation strategy to maintain cardiac output, calculated as the product of heart rate and stroke volume (Withers and Hillman 2001). However, this reallocation is highly costly (Costa et al. 2008), and any additional energy expenditure to regulate heart rate can substantially affect tadpole performance in other functions, such as swimming and foraging, particularly during early developmental stages (Gonçalves et al. 2024). Nevertheless, only the two DBH formulations affected this parameter, possibly reflecting the influence of other ingredients present in these commercial products. Although considered inert, these ingredients are trade secrets and are often more harmful than the active ingredient (Straw 2024). Heart rate was the only parameter in this study not affected by D_AI_, suggesting that the observed cardiotoxicity is likely associated with unreported ingredients in the commercial formulations, as discussed further below.

Exposure to D_AI_, DBH_1_, and DBH_2_ induced irregular swimming activity in tadpoles. Since exposure occurred during the transition to a free-swimming, feeding larval stage (stages 21–25, McDiarmid and Altig 1999), such behavioral alterations may compromise survival and fitness throughout development. These changes in swimming patterns could result from an energy reallocation triggered by 2,4-D exposure (Freitas et al. 2019). The increased incidence of lethargic tadpoles, in particular, may lead to ecological consequences, including reduced foraging efficiency, impaired predator avoidance, and limited habitat exploration, ultimately resulting in developmental delays and heightened predation risk (Santos et al. 2025). Altogether, the observed alterations in swimming behavior suggest a neurotoxic effect, corroborating studies that have demonstrated the ability of 2,4-D to affect both the central nervous and cardiovascular systems.

In addition, our results indicate a compromise of the antioxidant defense system, with effects modulated by the herbicide formulations. In the case of D_AI_, the decrease in catalase activity likely led to the accumulation of H₂O₂ in cells, potentially resulting in the formation of highly reactive and harmful free radicals, such as the hydroxyl radical (•OH) (Hidalgo et al. 2020). This mechanism explains the oxidative damage detected through changes in TBARS levels, a marker of lipid peroxidation. The observed pattern, with a decrease in TBARS at 5 µg/L and an increase at 10 µg/L of D_AI_, represents a classic hormetic response. Similar non-linear responses were observed in survival and heart rate, although the magnitude of these effects varied across concentrations. At the biochemical level, at low doses, the mild stress induced by D_AI_ may have compensatorily activated other defense pathways, reducing damage below basal levels (Calabrese and Mattson, 2023). However, as the concentration increases, these defense mechanisms are overwhelmed, and the inhibition of CAT at higher doses (20 and 25 µg/L) leads to increased lipid damage. This membrane damage can, in turn, alter the activity of other enzymes in the antioxidant system and potentially cause DNA damage (Halliwell et al. 2021), resulting in widespread oxidative stress. Such oxidative damage may underlie the physiological and behavioral effects observed in this study, including changes in heart rate and swimming behavior, which have also been reported in other amphibian studies (Costa et al. 2008; Pompermaier et al. 2024).

When analyzing the effects of DBH_1_, this formulation appeared to potentiate the pro-oxidant effect of the active ingredient. The significant increase in TBARS levels at 15 µg/L, without the protective phase observed with D_AI_, suggests that the adjuvants present in this formulation enhance the bioavailability or toxicity of 2,4-D. This may overwhelm detoxification systems, leading to an oxidative stress peak at an intermediate concentration (Eskander and Saleh 2020).

Finally, DBH_2_ reveals additional complexity. Despite decreasing CAT activity at all concentrations tested, DBH_2_ did not elevate TBARS levels; in fact, a reduction was observed at 5 µg/L. This response suggests that the adjuvants in DBH_2_ not only modulate but fundamentally alter the organism’s response to the herbicide. The observed CAT inhibition indicates the presence of a component in the formulation that acts as a potent inhibitor of this enzyme (Calabrese and Kozumbo 2021; Chivu et al. 2025). The reduction in TBARS, particularly at the lowest concentration, may be explained by a compensatory activation of other antioxidant systems, which temporarily mitigated lipid damage (Zeng et al. 2025). However, the persistent inhibition of a primary defense enzyme such as CAT leaves the organism more vulnerable to additional environmental stressors, representing a significant ecological risk even in the absence of acute lipid peroxidation.

The differential effects observed between the active ingredient (D_AI_) and the commercial formulations (DBHs) may be attributed to differences in product composition. In addition to the active ingredient, DBHs contain various co-formulants—such as stabilizers and solubilizers—that can influence toxicity (Seralini 2024). The type and concentration of these adjuvants vary widely among formulations and may include potentially toxic residual compounds (Mesnage et al. 2013). Such ingredients, or co-formulants, are not subject to specific toxicological evaluations, which increases uncertainty regarding the safety of commercial formulations (Seralini 2024). Research on inert ingredients has primarily focused on their interactions with the active ingredient to ensure they do not compromise efficacy against the target organism (Nagy et al. 2020). However, scientific evidence, including findings from our study, underscores the need to investigate the effects of these ingredients in isolation, as their ecological fates are unlikely to be uniform across an entire formulation (Straw et al. 2022). Additionally, factors such as industrial secrecy and frequent changes in composition limit transparency regarding the components used (Defarge et al. 2018). The differences in lethal and sub-lethal effects observed between the DBHs may therefore result from qualitative and quantitative variations in these inert ingredients, given that the concentration of 2,4-D is the same across all formulations.

Given that commercial formulations of 2,4-D are widely used in Brazil and other countries (Souza et al. 2023), tadpole populations inhabiting areas near agricultural zones may be at risk of decline. In extreme cases, this could lead to the local extinction of more sensitive species (Ferrante et al. 2019). Furthermore, they may experience sublethal effects, such as cardiotoxicity and irregular swimming activity, as observed in this study. These physiological and behavioral impairments could potentially reduce the ecological performance of these organisms, which may in turn affect their roles within the community and consequently alter population dynamics and trophic interactions in the aquatic ecosystems of these regions (Ascoli-Morrete et al. 2022).

These findings underscore that the effects of 2,4-D are strongly influenced by its final formulation. While the active ingredient exhibits a relatively predictable dose-response pattern, commercial formulations can both enhance its harmful effects (DBH_1_) and introduce additional mechanisms of action, such as selective enzyme inhibition (DBH_2_), which may mask or modify the ultimate manifestations of oxidative stress. Overall, our results highlight the critical need for environmental risk assessments of herbicides to evaluate not only the active ingredient but also the commercial formulations in their entirety.

Based on these parameters, it can be concluded that both D_AI_ and the DBHs affected the evaluated endpoints in *P. gracilis* at environmentally relevant concentrations, however, DBH_2_ was the formulation that most affected the early life stages of these animals. This shows that 2,4-D at low, environmentally relevant concentrations has harmful effects on this species and that, depending on the formulation used, these effects can be even greater, affecting the survival, physiology, and behavior during the early life of these animals.

## Conclusion

Our study expands the knowledge on the toxicity of both the active ingredient and commercial formulations of the herbicide 2,4-D to non-target organisms, using early life stages of the native species *Physalaemus gracilis* as an experimental model. The concentrations evaluated fall within the legally permitted limits in Brazil; nevertheless, they induced physiological, behavioral, and biochemical alterations in exposed tadpoles. These findings demonstrate that concentrations currently considered safe by legislation may, in fact, compromise biodiversity and the perpetuation of native species.

This study therefore highlights the need for additional research that systematically compares the effects of different pesticides and their commercial formulations with their respective active ingredients, in order to support the regulatory assessment of these widely available compounds. Furthermore, it underscores the importance of raising awareness and implementing measures to reduce pesticide use, particularly for those already known to be harmful to wildlife, as demonstrated here for 2,4-D exposure during early amphibian development.

## Supplementary Information

Below is the link to the electronic supplementary material.

Supplementary Material 1

## Data Availability

The datasets used and/or analysed during the current study available from the corresponding author on reasonable request.
