# Supplementary material for: Effects of the herbicide 2,4 D active ingredient and commercial formulations on the early life stages of the amphibian Physalaemus gracilis
Source: Ecotoxicology. 2026 Jun 17;35(5):117. doi: 10.1007/s10646-026-03094-9 (PMC13275561; doi:10.1007/s10646-026-03094-9)
Supplement: Supplementary file 1 — Supplementary Material 1 [file 10646_2026_3094_MOESM1_ESM.docx]

**Supplementary Material**

Title: Effects of the herbicide 2,4 D active ingredient and commercial formulations on the early life stages of the amphibian *Physalaemus gracilis*

Cristina Bridi, Guilherme Ferreira Felicioni, Jenifer Eduarda Luterek, Inete Cleide Baú, Flavia Bernardo Chagas, Aline Pompermaier, Carla Alves, Paulo Afonso Hartmann, Marilia Hartmann.

This supplementary material contains additional tables supporting the results of the main article.

Table 1 - Mean and standard deviation of heart rate for all products and concentrations tested. Active ingredient (D_AI_); Commercial formulation 1 (DBH_1_); Commercial formulation 2 (DBH_2_).

| 2.4-D | Group (µg/L) | Mean | Standard deviation |
| --- | --- | --- | --- |
|  | C | 128 | 24.39 |
| D_AI_ | 5 | 132.4 | 11.76 |
|  | 10 | 126.8 | 25.73 |
|  | 15 | 143 | 22.77 |
|  | 20 | 128.8 | 9.62 |
|  | 25 | 133 | 14.15 |
| DBH_1_ | 5 | 121 | 10.51 |
|  | 10 | 132 | 14.72 |
|  | 15 | 103 | 14.05 |
|  | 20 | 122 | 9.64 |
|  | 25 | 94 | 22.21 |
| DBH_2_ | 5 | 176.89 | 12.16 |
|  | 10 | 182.89 | 16.40 |
|  | 15 | 151.25 | 24.50 |
|  | 20 | 158.67 | 26.86 |
|  | 25 | 170.93 | 25.59 |

Table 2 - Mean and standard deviation of *Physalaemus gracilis* tadpoles exhibiting irregular swimming activity after seven days of exposure to the active ingredient and two commercial formulations of 2,4-D. Active ingredient (D_AI_); Commercial formulation 1 (DBH_1_); Commercial formulation 2 (DBH_2_).

| 2.4-D | Group (µg/L) | Mean | Standard deviation |
| --- | --- | --- | --- |
|  | C | 0.111 | 0.46 |
| D_AI_ | 5 | 0.883 | 0.99 |
|  | 10 | 0.583 | 0.70 |
|  | 15 | 0.757 | 0.86 |
|  | 20 | 0.714 | 0.04 |
|  | 25 | 0.871 | 1.01 |
| DBH_1_ | 5 | 0.794 | 0.94 |
|  | 10 | 1.045 | 0.89 |
|  | 15 | 0.750 | 0.93 |
|  | 20 | 0.649 | 0.49 |
|  | 25 | 0.578 | 0.50 |
| DBH_2_ | 5 | 1.227 | 1.02 |
|  | 10 | 1.366 | 1.17 |
|  | 15 | 1.364 | 1.20 |
|  | 20 | 1.148 | 1.13 |
|  | 25 | 1.882 | 1.26 |

Table 3 - Mean and standard deviation for TBARs levels for all products and concentrations tested.

| 2.4-D | Group (µg/L) | Mean | Standard Deviation |
| --- | --- | --- | --- |
|  | C DMSO | 0.0001424 | 2.591e-005 |
| D_AI_ | 5 | 8.120e-005 | 5.971e-005 |
|  | 10 | 0.0002114 | 1.232e-005 |
|  | 15 | 0.0001526 | 4.369e-005 |
|  | 20 | 0.0001197 | 1.050e-005 |
|  | 25 | 0.0001129 | 3.064e-005 |
|  | C | 0.0002424 | 6.582e-005 |
| DBH_1_ | 5 | 0.0003130 | 6.813e-005 |
|  | 10 | 0.0002405 | 0.0001120 |
|  | 15 | 0.0006370 | 0.0007258 |
|  | 20 | 0.0001880 | 0.0001536 |
|  | 25 | 0.0003073 | 8.856e-005 |
| DBH_2_ | 5 | 3.512e-005 | 4.885e-006 |
|  | 10 | 0.0001507 | 0.0001069 |
|  | 15 | 0.0001949 | 0.0001986 |
|  | 20 | 0.0001324 | 9.751e-005 |
|  | 25 | 0.0001201 | 4.366e-005 |

Table 4 - Mean and standard deviation for CAT levels for all products and concentrations tested.

| 2.4-D | Group (µg/L) | Mean | Standard Deviation |
| --- | --- | --- | --- |
|  | C DMSO | 0.1055 | 0.08565 |
| D_AI_ | 5 | 0.08245 | 0.1090 |
|  | 10 | 0.1900 | 0.1661 |
|  | 15 | 0.06849 | 0.06012 |
|  | 20 | 0.01676 | 0.01344 |
|  | 25 | 0.01002 | 0.003787 |
|  | C | 0.1149 | 0.06405 |
| DBH_1_ | 5 | 0.09601 | 0.05653 |
|  | 10 | 0.09672 | 0.08193 |
|  | 15 | 0.1032 | 0.07656 |
|  | 20 | 0.1076 | 0.06233 |
|  | 25 | 0.1293 | 0.06284 |
| DBH_2_ | 5 | 0.02310 | 0.01611 |
|  | 10 | 0.02424 | 0.01477 |
|  | 15 | 0.04568 | 0.05184 |
|  | 20 | 0.01462 | 0.01326 |
|  | 25 | 0.01931 | 0.009141 |
